# Negotiating the turning point in the transition from curative to palliative treatment: a linguistic analysis of medical records of dying patients

**DOI:** 10.1186/s12904-020-00602-4

**Published:** 2020-06-26

**Authors:** Laila Hov, Oddgeir Synnes, Guri Aarseth

**Affiliations:** 1 VID Specialized University, Diakonveien 14, 0370 Oslo, Norway; 2 Larvik Municipality, Sentrum Legekontor, Sigurds gate 4, 3256 Larvik, Norway

**Keywords:** Medical records, Dying patients, Palliative care, Linguistic analysis, Hospitals

## Abstract

**Background:**

Many deaths in Norway occur in medical wards organized to provide curative treatment. Still, medical departments are obliged to meet the needs of patients at the end of life. Here, we analyse the electronic patient record regarding documentation of the transition from curative to palliative care (i.e. the ‘turning point’). Considering the consequences of these decisions for patients, they have received surprisingly little attention from researchers. This study aims to investigate how the patient record denotes reasons for the shift from curative treatment to palliation and how texts involve voices of the patient and their families.

**Methods:**

The study comprised excerpts from electronic patient records retrieved from medical wards in three urban hospitals in Norway. We executed a retrospective analysis of anonymized extracts from 16 electronic patient records, searching for documentation on the transition from curative to palliative care.

**Results:**

In the development of the turning point, the texts usually shift from statements about the patient’s clinical status and technical findings to displaying uncertainty and openness to negotiation with different textual voices. This shift may represent a need to align or harmonize the attitudes of colleagues, family, and patient towards the turning-point decision. The patient’s voice is mostly absent or reported only briefly when, in their notes, nurses gave an account of the patient’s opinion. None of the physicians’ notes provided a detailed account of patient attitudes, wishes, and experiences.

**Conclusion:**

In this article, we have analysed textual representations of patient transitions from curative to end-of-life care. The ‘reality’ behind the text has not been our concern. As the only documentation left, the patient record is an adequate basis for considering how patients are estimated and cared for in their last days of life.

## Background

A significant proportion of patients receive end-of-life care and die in hospitals [1–3]. In the United Kingdom, 50% of all deaths occur in hospitals, and in France, the number is 57.1% [4, 5]. In Norway, which is the context of this study, 30% of all deaths in 2016 took place in hospitals [6]. Due to the ageing population, the complexity of chronic illness, and a growing number of people living alone, the number of hospital deaths will likely increase in the future [2]. According to the European Association of Palliative Care, palliative care takes a holistic approach, addressing physical, psychosocial, and spiritual care, including the treatment of pain and other symptoms [7], and should also be offered in hospitals [8]. Two systematic reviews investigating which elements patients and their families describe as important in end-of-life care in hospitals found that effective communication and shared decision-making were important. In addition, expert care, respectful and compassionate care, trust and confidence in clinicians, an adequate environment for care, minimizing the burden and family involvement, and financial affairs were important [2, 9]. An ability to maintain a sense of self was particularly essential to the patients. Family members highlighted the maintenance of patient safety, preparation for death, care being extended to the family after the patient’s death, and patient choice at the end of life [9]. These elements are in line with what Walter [10] describes as characteristics of good ways of dying in our time: ‘Open communication, choice, control, and a natural accompanied death’.

Kaufman states that death in the context of medicine is under constant negotiation and asserts that the main challenge revolves around the fact that modern medicine makes it possible to ‘time’ death [11]. The focus on the timing of death is reinforced by the infrastructure in hospitals, where technical equipment, interventions, and numerous medications are used to delay death for as long as possible [11, 12]. Access to advanced high-tech, life-extending medicine complicates decisions to stop treatment and transition to palliative care. Here, another essential element is patients’ and families’ contradictory feelings around ‘living as long as possible’ and a ‘good death’ [11].

An additional aspect of the modern hospital is the now well-established system of electronic documentation. The electronic patient record (EPR) is an essential part of clinical practice, and health care professionals are obliged by law to document relevant and necessary information about the patient and the patient’s care [13]. In the Western world, the past 20 years have seen a transition from patient records written on paper to EPRs [14, 15]. In Norway, this development is part of a more extensive process aimed at digitalizing patient records—the goal of Norway’s one patient–one record [16], which seeks to ensure that all involved health professionals are up-to-date across all concerned health care institutions and includes easier patient access [16].

Against the backdrop of our interest in the conditions for dying patients in hospital and EPR documentation in medical wards, our approach is the textual presentation of the transition from curative or life-prolonging care to palliative care that takes place shortly before the patient dies. We investigated what we refer to as the ‘turning-point note’. The turning-point note refers to the moment when a physician documents the decision to change the treatment aim from curative or life-prolonging to palliative care. We used the concept of a turning point because it illustrates the significant transition from a focus on saving the patient to a focus on relieving the patient’s symptoms and accepting death as the most probable outcome. As conflicting interests and difficulties regarding when to stop treatment often arise in hospitals around the context of dying patients, we investigated how these issues are reflected in the EPR. Our primary research question was, ‘How does the textual voice argue for the shift from curative to palliative care, and what characterizes the language that documents the interaction with the patient and family (i.e. external voices) towards the turning point?’

### Previous research

Prior [17, 18] stated that the most common approach in field document research has primarily been to view documents as sources of information that mirror the events that took place. Our search for literature confirms this. Goodlin [19], Shea [20], and Solloway [21] showed that decisions about the level of treatment and place of death affect both the type and the aggressiveness of care. In a comparison between age groups, Rashidi [22] showed that older patients were hospitalized for fewer days and received less parenteral palliative medication at the end of life.

However, we also found studies that pointed to deficits in the medical record; Parish [23] found a lack of appropriate assessment of and documentation about physical, psychosocial, and spiritual care at the end of life. Gholiha et al. [24] investigated patient records at the end of life and found a lack of documentation about physicians’ communication with the dying patient regarding the situation. Fins et al. [25] used the methodology of narrative ethics when they invited health care professionals to reflect upon the content, but not the linguistics, in how the last days of life were described in the medical record. They found that the structure of the notes ‘may reduce the opportunities to record the nuances of patient care’. A systematic review by Huber thematizes how the EPR can be used to facilitate the documentation of advanced care planning [26].

To our knowledge, little previous research has investigated *how* the documentation is written in the last days of life, in any context. Alternatively, however, documents can be seen as data in their own right, not as reports of reality but as producers of reality. A relevant study exploring the effect of language in this context was conducted by Aarseth et al. [27–30], who used a narratological and linguistic approach to analyse medical certificates of disability benefits written by general practitioners. They found a symptom-oriented focus in which, the authors argued, the patients were passive objects. Two other studies also approached the text as an active agent that construed its own reality: Engebretsen’s [31, 32] analysis of child welfare records and Aaslestad’s research on more than a century’s worth of medical records from a psychiatric hospital [33]. Because our study focused on how the decision to stop curative treatment is expressed and argued for through professional language in medical texts, we also approached the documentation as both situated and productive within a specific context rather than as a fixed source of information [24, 31].

## Method

The design used was a qualitative text analysis based on a theoretical framework where we explored the language as dialogic [34, 35]. We drew upon Bakhtin’s concept of the ‘dialogic language’ [34, 35]. The essence of Bakhtin’s theory is that an utterance can only make sense when it is spoken/written as a response to a previous utterance, or when it anticipates a response from others [35]. Our theoretical approach then, rested on an understanding of EPR text as a social actor that reflects, construes, and reproduced reality, working to induce an intended effect [17, 27, 31]. The work of JR Martin and PRR White [36] has also proved helpful, particularly their understanding—with reference to Bakhtin’s concept—of *all* utterances as dialogic (monologues included). With that, Martin and White expanded Bakhtin’s dialogic concept by stating that utterances are not either monologic or dialogic but are *more or less* dialogic. Furthermore, they view utterances expressing uncertainty or subjectivity as dialogically expansive—that is, open to alternative voices. Conversely, absolute statements are dialogically contractive in that they ignore, suppress, or reject alternative voices. Finally, we used specific elements from Martin and Rose’s linguistic framework of appraisal (i.e. affect, judgement, and appreciation) to investigate utterances of appraisal and, thus, the interpersonal aspects of language [37].

Anchored in these analytical approaches, we used their suggested analytical tools to explore how the textual voice positions itself towards external voices: aligning with them, opposing them, or taking a neutral or undecided stance. Also, we sought to understand how the textual voice relates to the reader (for example, does the textual voice expect that the reader will find it doubtful, problematic, or commendable?). In doing this, we chose to distinguish between the textual voice and the health professional (the person who actually wrote the notes). This distinction implies that the text might represent values and attitudes that are not necessarily representative of the writer’s personal values [31]. Throughout our analysis, when we use the term ‘reader’, we refer to the intended readers who have access to the EPR documentation—usually colleagues, patients, and their families, but also the possible ‘super-addressees’, the supervisory authority whom the text does not address, but whom it is aware of as a possible reader [31].

### Data collection

The EPRs were retrieved from urban hospitals in specialist health care services in Norway. Seven hospitals were invited to participate, and three accepted the invitation. Protection offices at the hospitals assessed and approved the project before our contacts began selecting EPRs from the medical wards that were not particularly adjusted to palliative care patients. Our contacts were then instructed to include patients from a list of the most recently deceased in the EPR system, following the inclusion criteria.

The inclusion criteria stipulated that records could only be selected where 1) the patient was deceased, 2) the patient was 18 years or older, and 3) the case summary written by the physician following the patient’s death noted that death had been imminent.

Moreover, to ensure anonymity, the patient ages were categorized into five-year intervals. We requested information from the patients’ final three to 6 days of life. Data collection took place between March and June of 2017. Our contacts at the participating hospitals printed out the their selected EPR excerpts and anonymized all personal information, diagnosis codes, ward information, and names of the health care professionals who had completed the documentation. We also instructed our contacts to fill out a form with the following information about each patient represented in the selected excerpts: 1) gender, 2) age, and 3) whether the patient was admitted to hospital from home, a nursing home, or another hospital. We recorded this demographic data, the length of the excerpts, and the number of days each represented.

Furthermore, we noted any do-not-resuscitate orders and when they were made, as well as any decisions to withdraw life-prolonging treatment. Part of our inclusion criteria was that the EPR had to contain an explicitly noted turning point leading to apparent changes to the patient’s treatment trajectory. Relevant changes would be a cessation of life-prolonging medications, cancellation of examinations, and surveillance of bodily functions. Simultaneously, palliative measures like administration of medication for symptom alleviation were added. Out of the 42 total EPRs, 16 contained a turning point and constituted the material for this study.

### Data analysis

All the included hospitals in this study used an EPR system called distributed information and patient data system (DIPS) for hospitals. DIPS is the most common system for EPRs in Norwegian hospitals. In DIPS, each group of health professionals has access to different types of templates, depending on their profession.

It was mainly physicians and nurses who wrote the notes, but in some of the cases, other professionals, such as physiotherapists or nutritionists, also wrote notes. All notes were saved in the patient’s file, but each profession had its folder, and all health care workers included in the patient care could access the documentation of others.

We conducted a textual analysis of the 16 EPR excerpts, the first step of which involved extracting all documentation providing information on the course of events leading to the turning point.

Additionally, to get an understanding of how the patients’ last days elapsed, we mapped the examinations, tests, treatments, and transferrals between wards that took place in the period leading up to the turning point. Regarding the documented interaction between health care professionals and family, all references made to the patient’s utterances, attitudes, and the family were analysed. Furthermore, we also gathered information that could shed light on the patient’s mental condition, because the mental condition indicates whether or not the patient was capable of having an opinion about the turning point. Through this mapping, we found that only documentation completed by a nurse or physician contained information relevant to the turning point.

We then conducted an in-depth analysis of the collected information. The first and last author of this article read and discussed the 16 cases in collaboration and developed preliminary categories. Following this step, we performed an exhaustive reading of each of the 16 cases to ensure that all relevant information was selected. Next, we refined the initial categories into meaningful themes that illuminated textual features pertinent to our research question [17].

Martin and White’s appraisal theory proved a useful analytical tool for identifying and analysing the evaluative language and dialogic style in the texts [35]. Furthermore, Halliday’s [38, 39] model of language as a representation of social meaning guided us in deciding who the participants were and what processes led up to the turning point.

We arranged regular meetings in addition to frequent email exchanges in order to ensure an accurate understanding of the text. Two of the three researchers (LH and GAA) were health care professionals themselves (nurse and physician), with experience in both writing and reading medical records.

### Ethical considerations

The regional ethics committee in Norway approved an exemption from the Duty of Confidentiality for a third person (referred to in this paper as ‘contact’), who was employed at the involved hospitals and had access to the documentation system to retrieve and anonymize the included EPRs (2016/2035/REK) before the researchers could access the documents. Permission to access the EPRs was obtained through correspondence with hospital management.

## Results

Our analysis was based on the 16 EPRs that contained explicit documentation on the transition from curative/life-prolonging treatment to palliative care (what we refer to as the turning point). We found that it was only in the physicians’ and nurses’ notes that the topic of the turning point was present. The age ranges of these 16 patients at the time of death were from 31 to 35 to 96–100, but almost all of the patients were over 66 years of age. The excerpts to which we had access covered a period of three to 6 days before death; the turning-point decision was made between 5 days and only a few hours before death. Eleven of the patients were admitted directly from their homes, while in four of the cases, the admission was from a nursing home, and in one case, the patient came from another hospital. Most of the patients died from organ failure; other causes of death were cancer, infections, and internal bleeding (cerebral and gastrointestinal). In general, the level of treatment activity remained high until the turning point. Two patients received nutritional support, and three patients were transferred to intensive care before the turning-point decision. Three patients received non-invasive ventilation, and nine patients were treated with antibiotics. An X-ray of the thorax was taken for seven patients. In comparison, three patients had computerized tomography (CT), and one had a colonoscopy. There were also two gastroscopies in one patient, and another patient had revision surgery (see Table 1 for an overview of the treatments in the included EPRs).

**Table 1:** Demographics and clinical features of the 16 included medical records

Patient No.	Length of excerpt (days)	Gender	Age (5-year interval)	Cause of death	Admitted from	Blood tests	Antibiotics	Other acute medication	Nutritional support	Examinations	NIV	Transferrals	Discharge planning	Time of death after TP
1	4	M	71–75	Pulmonary failure	NH	Yes	Yes	–	–	–	CPAP	–	–	3 days
2	5	F	31–35	Liver failure	Home	Yes	–	–	Tube nutrition	X-ray	–	–	–	2 days
3	5	M	91–95	Sepsis	NH	Yes	Yes	–	–	CT	–	Medical ward to ICU	Planned return to NH	3 days
4	5	M	76–80	Pulmonary oedema	Home	Yes	–	–	–	X-ray, CT	–	–	Planned discharge to NH	1 day
5	3	M	81–85	Pneumonia	Home	Yes	Yes	–	–	X-ray	BiPAP	Dies in ICU	–	Hours
6	4	F	76–80	Cancer/heart failure	Home	–	–	–	–	Ultrasound	–	–	Planned discharge to NH	Hours
7	5	F	81–85	COPD/pulmonary oedema	NH	Yes	Yes	Diuretics, nitroglycerin	–	–	VPAP	–	–	Hours
8	4	F	66–70	Cancer	Home	Yes	Yes	Blood, albumin	–	Revision surgery	–	–	Planned discharge to NH	Hours
9	5	M	86–90	Parkinson’s disease	NH	–	–	–	–	–	–	–	–	5 days
10	4	F	81–85	Heart failure	Home	Yes	Yes	Antiarrhythmicum	–	X-ray	–	ICU to medical ward	–	1 day
11	4	F	86–90	Infection/cancer	Home	Yes	Yes	–	–	X-ray, coloscopy	–	–	Planned return to NH	1 day
12	6	M	> 96	Heart failure	Hospital	Yes	–	–	–	X-ray, ECG	–	–	–	1 day
13	4	K	71–75	Cerebral haemorrhage	Home	Yes	–	Blood pressure medication	–	CT	–	Dies in OU	–	2 days
14	6	M	91–95	Multiple organ failure	Home	Yes	Yes	–	Intravenous nutrition	X-ray	–	–	–	1 day
15	3	M	76–80	Kidney failure	Home	Yes	Yes	–	–	–	–	–	–	Hours
16	4	K	91–95	Gastrointestinal haemorrhage	Home	Yes	–	–	–	Two gastroscopies, with a third planned	–	From ICU when TP	–	2 days

Note: *F* Female, *ICU* intensive care unit, *M* male, *NH* nursing home, *OU* observation unit, *TP* turning point, *X-ray* x-ray of thorax

**Fig. 1:**
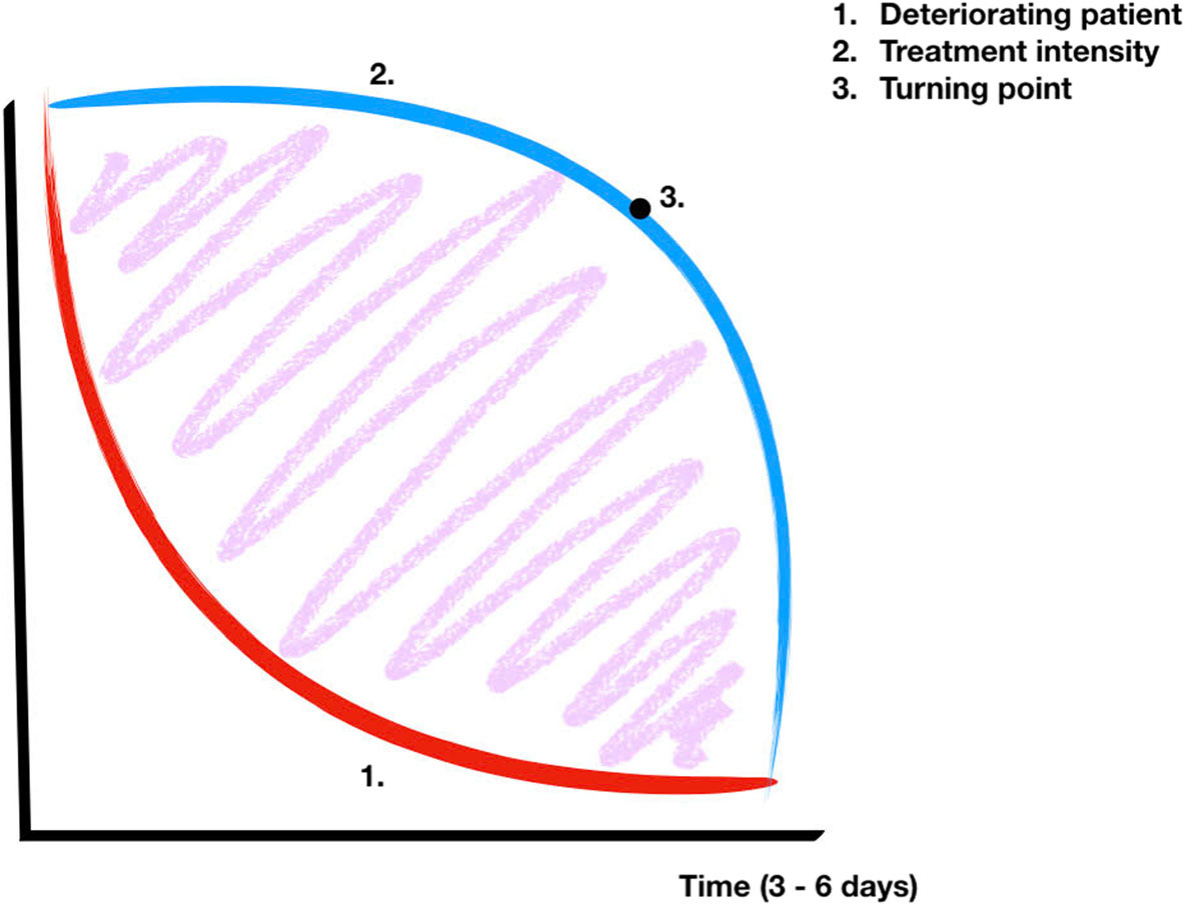
Visualization of intensity of treatment vs description of the deteriorating patient. Red line: patients’ clinical conditions. Blue line: treatment intensity.

In summary, most patients received a large number of medical interventions, medicines, and procedures until shortly before death—no stone seemed left unturned. Surprisingly, age did not appear to affect the number of interventions to which the patient was exposed. In Fig. 1, we outline the typical course in the days before the patient died. In essence, treatment intensity remained high until the turning point, and most of the patients died shortly after the decision was made.

We aimed to investigate the rhetoric of the textual voice leading up to the turning-point decision and what characterized the representation of the interactions with the patient and family when arriving at the turning point. We grouped our findings into three main themes:

Theme 1) Positioning of the textual voice: This varies within a scope of anonymous or formalistic distancing and subjective (personal?) responsibility.

Theme 2) Representations of the external voices: With few exceptions, the terminal patient’s attitudes to stopping curative treatment was poorly clarified and was inferred from the textual voice. Also, the physician’s uncertainty towards the turning point was displayed, involving the family, leaving the space open for their voices.

Theme 3) Subtle traces of disagreement and differing perspectives between professionals: Physicians’ voice and nurses’ voices tend to emphasize different aspects of the dying patient’s conditions, creating a professional gap.

### Theme 1) positioning of the textual voice

We investigated how the textual voice placed itself in the retelling of the events and found it was through one of two main strategies: 1) through the veiling of the speaking subject, or 2) through being more or less responsible for the utterances.

#### Veiling of the textual voice

There appeared to be three ways of veiling the textual voice: 1) removal of the speaking subject, 2) impersonalization of the speaking subject, and 3) use of the collective ‘we’.

One way of veiling is to eliminate the writer subject, as these examples show:

Indeed seems terminal and have assessed the need for relieving care alone. (Case 15, man, 76–80)

Think from an overall assessment that it is not right to transfer the patient to intensive care when she so clearly expresses that she wants to be spared. (Case 11, woman, 86–90)

Removing the subject from the sentence is a way of anonymizing who takes responsibility for the statement. However, in these examples, the first words are ‘seems’ and ‘think’—according to Halliday [38], these are psychological processes in the mind of the writer, anchoring the utterance in the writer’s contingent subjectivity [36] and thus confirming his/her responsibility for the assessment. The utterances express his/her professional and subjective stance and are therefore dialogically expansive, implicitly open to alternative voices to have their say on the matter.

Another way of veiling the writing subject is through the use of the impersonal pronoun ‘one’. For instance,

One assesses that one is at the end of the road. (Case 5, man, 81–85)

In this example, the double use of an impersonal pronoun refers to the assessor of the patient’s condition as well as the patient, resulting in an utterance where both the subject and the object are anonymized. Whether it is the physicians’ treatment options or the patient’s life that is reaching the ‘end of the road’ is unclear. The use of ‘one’ in the example above has further implications because the assessment is detached from the subjective responsibility of writing ‘I’, appearing to be based on a joint professional agreement. If ‘one’ also refers to the patient, it may be seen as a *de*personalization of the object. Likewise, a particular form of depersonalization is the metonymy that is using one entity to refer to another to which it is related [27, 40, 41]. In the following textual excerpt, ‘abdomen’ serves as a metonymy for the patient:

As a starting point, one does an ultrasound of the abdomen, from which one moves over to a CT, where one unveils a probable caecum cancer with carcinomatosis and metastasis to the lungs. Cessation of all treatment included fluids. (Case 3, man, 91–95)

This excerpt also shows a complete impersonalization of the writer to the extent that nothing in the text points to anyone’s responsibility for declaring the ‘cessation’.

Third, we found several examples in which the authorial voice is present through the plural pronoun ‘we’. By using ‘we’, the decision is presented as a collective process involving both the medical staff and the patient’s family (*‘Both we and the family feel that this is the right decision’* (case 2, woman, 31–35)), or the medical staff only *(‘If this does not change the situation we must end treatment’* (case 5, man, 81–85)).

#### The responsible textual voice

In the previous section, we showed ways of hiding the textual voice. However, we also found examples in which there was a distinct textual voice, albeit to a lesser extent. We found two ways that the textual voice takes explicit responsibility for the turning-point decisions. The following three examples show a clear textual voice—a speaking subject that contributes to the assessment of the patient.

In the first two examples, the textual voice argues as to why arriving at the turning point is a necessary step, but it does so in different ways.

If she gets worse with a continued fall in her general condition and dyspnoeic, I think one should aim for a palliative approach by first and foremost focusing on relieving the patient’s discomfort. (Case 10, woman, 81–85)

In this note, the textual voice is present through the use of ‘I’. However, by using ‘if’ and ‘one should’, the textual voice indicates a sense of personal reservation, leaving it open to both allow others to disagree with the opinion of the writer and also to the possibility of the patient getting better.

In the second example, the authorial voice is also present with an ‘I’:

I think it is not ethically right to go for active treatment now that the patient has a very low level of functioning with low quality of life and will become even more deteriorated out of hospital, with subsequent even lower quality of life and a probable short remaining lifetime. Consequently, I choose palliation. (Case 9, man, 86–90)

Relevant in this case is that the turning point was decided while the patient was still in the emergency ward. In this excerpt, the textual voice was aimed at convincing the reader that a transition to palliative care was the right decision by stating that the patient’s quality of life was low and that he only had a short time remaining. Simultaneously, through the use of ‘think’ and ‘probable’, the note is dialogically open to other opinions. Thus, the writer takes on the personal responsibility for the patient’s situation and actively ‘chooses’ palliative care, reinforced by the presence of ‘I’. However, at the same time, it communicates an openness for negotiation because a colleague could ‘choose’ differently.

In the third example, the textual voice is a specialist in palliative care who has consulted on a patient’s case:

Critical that her family receives information that we now assess her to be dying. I fear that she can die within the next 24 hours, in the worst case. (Case 8, woman, 66–70)

This example is an exception in the empirical material, as the textual voice expresses a feeling (‘fear’), which is unusual in the context of medical texts. The textual voice in this example expresses fear that the patient may die within the next 24 h. Why the expert in palliative medicine would ‘fear’ the patient’s death is unclear, but the intention might be to stress the importance of the information: is it ‘critical that her family receives information’, or is it a fear of her dying? Regardless, this example illustrates how unclear these texts can be and how open they are to different interpretations.

Finally, we highlight another interesting element in this category—the frequent use of ‘undersigned’. For example,

Undersigned informs that there is a potential to successfully treat his infection, but despite this, he does not wish for any more treatment. (Case 4, man, 91–95)

‘Undersigned’ presents the authorial voice as the responsible subject; however, it represents a more formal discourse, likely aimed at increasing professional authority and toning down personal involvement.

### Theme 2) representation of external voices

Now, we turn to our investigation of the external voices in the text. Here, ‘external voices’ refers to how the patient and the family are represented in the EPR.

#### Patient involvement

Patient involvement is emphasized as an essential topic in both palliative care and in legal regulations [42] for documentation in the medical record. However, the manner in which the patient/family voice was expressed in the material varied. We found the most pronounced patient voice in two cases: in both of these, the patient was resisting treatment and, through this, appears as an external voice in the text. In the first example, it is evident that the patient has chosen to go against the physician’s recommendation:

Undersigned informs that there is a potential to successfully treat his infection, but despite this, he does not wish for any more treatment. (Case 4, man, 91–95)

In the second example, the textual voice refers to the patient’s request to let her die because it has ‘been a lot’:

Says that she thinks there has been a lot now. Asks directly if we can let her die. (Case 11, woman, 86–90)

In both of these examples, the patient’s wish was used as the main argument, implying that the patient, as presented in the text, actively contributed to the turning-point decision.

However, these are the only examples in which the patient voice is weighted so heavily. In another instance, the rejection of treatment was presented as the patient’s refusal of food, and reference is made to the patient’s body language:

Offered food but did not want. Chews on the straw instead of drinking. Says stop and that he does not want. Shakes his head when I ask him about Kabiven [intravenous nutrition]. (Case 14, man, 91–95)

However, the descriptions of the patient’s rejection of food and dismissive body language are not a part of the turning-point decision:

Decides in deliberation with the family to end treatment and focus on alleviation. (Case 14, man, 91–95)

In one case, the textual voice concludes that

The patient expresses acceptance that the end is near. Has no pain or ailments. (Case 15, man, 76–80)

Here, the textual voice presents a summary of a conversation in which the patient has expressed that he accepts that he is dying. However, how the patient demonstrated this acceptance is not provided. Instead, the textual voice encloses the dialogue with the patient by stating he ‘has no pain or ailment’, which is a dialogically contractive statement. Through this statement, the authorial voice also implies that there is no need to focus on the patient’s pain and ailment because he has none.

In some cases, the patient is regarded as unable to be involved in the decision-making. By stating that the patient is too weak or disoriented, the textual voice disqualifies the patient from being involved in the process. Here, also, the writing subject is removed or impersonalized, leading to objectifying statements:

It cannot be done to discuss treatment level with the patient today. (Case 12, man, older than 96)

Due to (patient’s) general condition, one cannot discuss it with her. (Case 16, woman, 91–95)

In one example, we are introduced to a reflective patient and a textual voice that seems to praise the patient for having insight into her condition:

Very reflective regarding her disease situation. She seems prepared that it may go that way. At the same time, I sense a clinical improvement today and that she expresses a strong will to get through this if she can make it. (Case 7, woman, 81–85)

The patient is aware that she may die (‘seems prepared it may go that way’), which is followed by the textual voice, visible as ‘I sense clinical improvement’. In a professional context, the word ‘sense’ opens up to an interpretation that the textual voice empathizes with the patient and wants the patient to get better.

#### Family involvement

The patient’s family is more frequently involved than the patient as external voices in the representation of the turning point. In some cases, the family’s opinion becomes a part of the basis for the decision-making:

[Patient and family] agree not to examine liver finding further. (Case 6, woman, 76–80)

Sister with support of the family feels that the patient is not getting any better and that she suffers from ongoing measures. (Case 2, woman, 31–35)

Talked about treatment intensity with two sons. They do not want us to force food into him. Important that we treat him with a purpose, that there is something to treat him for, and that there is a chance for improvement. (Case 9, man 86–90)

In the last two examples, the reference made to the conversations with the family is through the use of indirect discourse. However, it seems uncharacteristic for the textual voice to use phrases such as ‘suffers from ongoing measures’ and ‘force food into him’, and we may presume that these are retellings of how the family expressed their opinion.

Here, the overall impression is that a primary aim of the text is to align the family in support, or at least acceptance, of the decision. The most significant finding regarding family involvement is the frequent use of the word ‘acknowledge’:

They acknowledge CPR [cardiopulmonary resuscitation] minus. (Case 4, man, 76–80)

Patient and family already acknowledge severe illness and prognosis pessima [poor prognosis]. (Case 6, woman, 76–80)

They seem to be acknowledging that we will not do CPR if something should happen. (Case 7, woman, 81–85)

Equivalent phrases in the excerpts include ‘take it calmly’, ‘shows understanding’, ‘feel that this is the right decision’.

We also found examples in which the family were passive recipients of the information. In these examples, the text is strictly informing the reader that the family knows that the patient is going to die:

Inform the sister about the decision to end treatment. (Case 1, man, 71–75)

The statement shows no interest in the sister’s reaction to the information and is not seeking her consent. The point is to inform the reader that the patient’s sister knows about the decision to end treatment.

#### Subtle language

A pervasive finding that applies to all of the above examples is the absence of the fact that the patient is dying. The only reference to death is when the patient raises the topic:

Says that she thinks there has been a lot now. Asks directly if we can let her die. (Case 11, woman, 86–90)

None of the other cases contain explicit reference to communication with the patient or family about what the turning point implies.

### Theme 3) subtle traces of disagreement and differing perspectives between professionals

Overall, the typical EPR text endeavours to present a picture in which all involved parties are on the same page. However, we found subtle traces of disagreement and differing perspectives between the professionals involved regarding the turning point. First, regarding traces of disagreement, we found two cases where the nurse, in the nurse’s documentation, indirectly expressed frustration with a lack of clarity around the patient’s level of treatment. In one example, the disagreement was communicated by using an exclamation mark:

Clarify treatment level tomorrow on the rounds! (Case 1, man, 81–85)

This makes the utterance appear as a command, and given the hierarchical structure in the hospital, the use of an exclamation mark turns this statement into an active request that articulates the nurse’s frustration with the situation. In another example, the nurse expresses different knowledge about the patient’s situation and indirectly instructs the physicians on behalf of the family:

The family does not want the staff to tell the patient that she is going to die. This also applies to the physicians on the rounds. (Case 2, woman, 31–35)

However, there is no reference to this topic in the corresponding physician documentation.

Second, nurses’ texts and physicians’ texts’ sometimes emphasized different perspectives, which is illustrated by the following excerpts, both concerning the same patient, and both written on the same day:

Physician: ‘Talked with patient and daughter, informed of findings of metastatic changes in the liver. . . . Heart failure must still be regarded as the clinical biggest challenge, with lack of response from diuretics and little to offer for additional treatment. . . . Wish not to expose the patient to unnecessary treatment. Agree not to examine liver finding further.’ (Case 6, woman, 81–85)

Nurse: ‘Pt. [patient] starts crying easily when she talks with u.s. [undersigned] about the fact that her husband and children will have a hard time when she is gone. Still, she is apparently aware that she is now so sick that she probably never will come home and that she does not have long left to live. U.s. perceives it as the patient is having a tough time accepting this and that she has no wish “to die before her husband, because he will be devastated”, according to the patient.’ (Case 6, woman, 81–85)

When comparing the information we are given from the nurse and from the physician, we see two very different stories being told. In the physician’s note, there is a veiling of the textual voice, which refers to a situation in which the patient and daughter have been informed of the patient’s status, then moves along to say, ‘wishes not to expose the patient to unnecessary treatment’. We do not know if this is the ‘wish’ of the physician or the daughter, but it seems like it is not the patient’s wish, since she is not referred to as an active participant in the conversation. In the nurse’s note, we are told of the patient’s grief and existential struggles. Here, the textual voice invites the reader into the conversation in which they talked about her feelings about being close to death. The use of indirect and direct discourse in this example displays the loss and grief felt by the patient.

Our results show that each of the 16 EPRs analysed argued (either explicitly or implicitly) that the turning point was unavoidable, but on various grounds. However, we found that these turning points more often than not were argued for through use of dialogically expansive language. This dialogical openness may reflect the level of uncertainty and complexity that is attached to these decisions. Our primary impression, which we detail in this discussion, is that turning-point arguments rooted in the patients’ experience of the situation are rarely foregrounded in the texts; instead, a focus on the lack of effect from treatment, the deteriorating body, aligning the family, and securing support for the decision are given priority in the text.

## Discussion

In this study, we have investigated how the EPR texts approach the shift from curative to palliative care at the end of life, here called the turning point.

All 16 of the EPRs argued (either explicitly or implicitly) that the turning point was unavoidable, but on various grounds. The medical and clinical reasoning preceding the turning point was, more often than not, a bare statement that was not open to alternative assessments. However, when approaching and justifying the turning point, the language became more dialogically open, in particular towards the patient’s family, which may reflect the uncertainty and ethical complexity that are attached to these decisions. Dialogic behaviour is needed to explore the attitudes of the patient’s family as a basis for negotiating consent about ending curative treatment. In contrast, turning-point arguments concerning the patients’ preferences and wishes are rarely foregrounded in the texts.

We also point to the invisibility of the speaker subject through the veiling of the authorial voice, using ‘one’ or ‘we’ instead of ‘I’. Impersonalization makes the text achieve two purposes: 1) It blurs the line of responsibility, meaning it becomes unclear as to who is responsible for the decision, and 2) it creates an impression that the turning-point decision is based on general agreement. Furthermore, when the external voice (i.e. the patient) is eliminated or presented as ‘one’, it appears as a downgrading of the patient subject that eliminates all of the patient’s personal features and has the effect of creating distance between the reader and the events that have taken place.

Second, the textual voice anticipates an intended reader who might either agree with or question the process leading to the turning-point decision. Consequently, various dialogical styles are in play regarding how open or closed the text is to alternative voices. When investigating the verbal processes [38, 39], we discovered the frequent use of terms that display a contingent subjectivity and, thereby, an openness that ‘allows’ for alternative opinions and value positions. As such, even though the turning-point notes in the 16 cases had in common that curative or life-prolonging treatment was to be abandoned, the turning point itself was more often than not written in a dialogically expansive language. This finding is surprising given that most treatment decisions up until the turning point are based on biomedical ‘facts’. An explanation for this sudden textual change with regard to alternative opinions could be a recognition of what is at stake for the writer, as the turning-point note holds much power over the trajectory of the patient’s last days. Accordingly, the writer becomes more engaged with a side gaze towards a super-addressee (for example, family, lawyer, supervisory authority) who is a potential reader of the text [31]. In consequence, the text shows that all parties could have their say in the matter, hence displaying that ‘everything proceeded according to standards’. However, while the text appears open to discussion, we found no opposing voices within the medical profession itself, and the only traces of dialogue between physicians was when the textual voice referred to a conversation with colleagues or used ‘we’ (and perhaps ‘one’). We have pointed to traces of disputes in the nurses’ documentation directed towards the physician(s). However, the physicians’ documentation is silent in response. We can thus only speculate whether this silence is because the physicians were unaware of the critique/request as they had not read the note, or whether it could also be that the textual voice is choosing to ignore the nurses’ frustration/suggestions. If the health professionals do not read documentation across their professional affiliation, it could jeopardize the collaboration about what would be in the patient’s best interest and be an obstacle for effective communication. Also, this would not be in line with the health care service’s overarching idea of ‘one patient–one record’ [16].

Third, there seems to be a clear tendency for the texts to construe harmony and alignment within and between the voices present, especially in the notes written by physicians. When the patient or family enters the text as either active participants who take part in the decision or passive participants informed of the decision in hindsight, the textual voice renders them as being at peace with the decision. Words describing their attitudes are [they] ‘acknowledge’, ‘take it calmly’, and ‘seem to understand’, whereas feelings of loss and grief are not described. The only exception to this was the nurse’s note about the woman who mourned her death on behalf of her family.

Similar to the use of harmonizing language, we wonder if a euphemism for death might serve the same purpose of leaving the difficult topics aside. This is in line with what Aaslestad found in his reading of psychiatric records when he wrote, *‘If the relation to dangerous topics becomes too persistent*. *.*. *the journal note answers with its safest grip: silence’* [33].

This textual analysis represents new and relevant information about the transition to palliative care in medical wards. To our knowledge, this is the first textual analysis that elucidates the internal and external voices in EPR documentation leading up to the decision to end curative or life-prolonging treatment. Our overall results indicate that in several aspects, EPR documentation reflects the main findings in Kaufman’s study: that modern medicine makes it possible to ‘time’ death [11]; the values promoted in the documentation are those dealing with death as a result of stopping treatment rather than as a natural outcome of living [11, 12]. In the analysed records, the main reason for ending curative or life-prolonging treatment was the failure of the treatment effect. That implies that trying out all possibilities was regarded as the default value unless the patient or family clearly expressed another opinion. The only exception we found was the turning point for which the textual voice stated, ‘I choose palliative care’. In that specific case, the textual voice argues that continuing treatment would be unethical.

The unilateral focus on ‘timing’ and ‘trying’, rather than on the patient’s situation, may partially explain the lack of a clear connection between recommendations and steering documents about palliative care [7, 8]. Moreover, the assumption that everybody wants to live as long as possible seems to be taken for granted in the medical context [11]. Hence, we believe that the need to argue for the turning-point decision implies an expected opposition that the textual voice must convince because the decision goes against the standard policy of trying all options.

It is easy to overlook the fact that all communication serves a purpose and thus makes it impossible to remain unpositioned [38, 39]. Our point of departure is that values infuse all texts [24, 43]. There is an expectation that medical documents will be impartial, objective, and neutral [44], and these values are taken for granted to the extent that it can be difficult to remember that they have been selected as core values for medical records. We might say the same regarding the value of ‘saving a life’ that we have shown to be omnipresent in the text. Moreover, the high level of repetition of phrases in the material indicates that the writing of the medical record is an automatized act that does not require much reflection. However, we argue that the golden standard of neutrality and objectivity [44] may justify a silencing of the individual patient experience. One consequence of this absence of the patient’s experience is that it contributes to maintaining a norm in which fear, grief, and mourning are devalued as subject to medical care, which may repress the holistic perspective that constitutes the core of palliative care [7, 8].

In our study, the primary issue centred around the treatment and when to stop. When the text works as intended, meaning promoting the best interests of the patient, all is well, but what happens when it does not? It may seem that the need for objectivity has led to the omission of information that would be valuable for the physician. The clearest example of this from our study is when ‘one’ is used to define both the writer and the patient. In these cases, aiming for neutrality makes the text blurry and vague and interferes with medical record standards of accuracy.

### Discussion of method

The core criteria for inclusion was an immediate change in the treatment trajectory after the turning-point note. The reason for this benchmark was the necessity of knowing that health care professionals acknowledged that death was a probable outcome by providing palliative care. Given that death in all of the cases was expected, how was dying approached in the excluded 26 EPRs? In a few, the patient had been hospitalized for longer than the excerpt period that we had access to. However, in most of the cases, there was a great variety in how the patients approaching death were represented in the text. In most of the cases, there was a mention of poor life expectancy (weeks or months), but no decision was made to stop curative or life-prolonging treatment. On the other side of the spectrum were the cases with no mention of prognosis and the patient receiving curative treatment until the time of death. However, with a few exceptions, the patient had a do-not-resuscitate and if resuscitation was initiated, a decision to stop was made shortly after starting cardiopulmonary rescue.

The data we used in this study were generated for purposes other than research, and as such, were obtained ready-made [30] and uninfluenced by the researchers [45]. The data selection, however, was based on the researchers’ interpretation of the texts, and we are aware that other researchers might interpret them differently.

The essence of textual analysis is to make the text speak [32], and the interpretation of meaning is a significant aspect of examining the text. Our interpretation was guided by our chosen theoretical lens and is one of many possible ways to approach the texts. In addition, our readings of the texts were influenced by the clinical backgrounds of two of the researchers (LH and GAA) as health professionals. We believe that this widened our understanding of both professional terms and the communication.

Regarding the generalizability of the study [45], we believe that studies using the same textual analytical tools on the same type of material will have similar findings. We also believe that our results are transferrable to EPRs from similar institutions. The reason for this assertion is that we retrieved the data from 11 different medical wards across three separate hospitals, and the similarities in the manner of writing styles were striking. We have reason to assume that our material spotlights how documenting the turning point is established across hospitals in Norway and maybe in other countries with similarly structured health care services.

## Conclusion

The present study, to our knowledge, is the first to perform a textual analysis of medical records at the end of life in medical wards. We do not attend to the external reality but only to the reality produced in the texts. When the EPR text mirrors the transition to palliative care, in which the turning-point argumentation primarily revolves around practicalities and the need to harmonize and align the participants in the text, we believe that relevant information about the patient’s experience and quality of life is lost. A text less neutral in its descriptions of events would provide more information that, in turn, would make it easier for the reader to be aware of the individual patient’s needs.

## Data Availability

The data that support the findings of this study are not available due to legal and ethical constraints imposed by the Regional Ethics Committees for Medical and Health Research Ethics in Norway.

## Abbreviations

CT: Computerized tomography
EPR: Electronic patient record
ICU: Intensive care unit
NH: Nursing home
OU: Observation unit
Pt.: Patient
TP: Turning point
U.s.: Undersigned
